# Ultrasonic quantification of cerebral perfusion in acute anterior circulation occlusive stroke—A comparative challenge of the refill- and the bolus-kinetics approach

**DOI:** 10.1371/journal.pone.0220171

**Published:** 2019-08-15

**Authors:** Jens Eyding, Raluca Reitmeir, Markus Oertel, Urs Fischer, Roland Wiest, Jan Gralla, Andreas Raabe, Irena Zubak, Werner Z´Graggen, Jürgen Beck

**Affiliations:** 1 Department of Neurology, University Hospital Knappschaftskrankenhaus Bochum, Bochum, Germany; 2 Department of Neurosurgery, Inselspital, University Hospital Bern, Bern, Switzerland; 3 Department of Neurology, Inselspital, University Hospital Bern, Bern, Switzerland; 4 Department of Neuroradiology, Inselspital, University Hospital Bern, Bern, Switzerland; University of Warwick, UNITED KINGDOM

## Abstract

**Purpose:**

To prospectively evaluate the potential of semi-quantitative evaluation of cerebral perfusion in acute ischemic stroke by comparing two established ultrasound approaches.

**Materials and methods:**

Consecutive inclusion of patients with acute occlusion of middle cerebral artery (MCA) confirmed by either magnetic resonance imaging (MRI) or computed tomography (CT) perfusion imaging qualifying for interventional therapy. Comparison of bilateral high mechanical index (MI) bolus-kinetics (HighMiB) and unilateral low MI refill-kinetics (LowMiR) performed before specific treatment.

**Results:**

In 16/31 patients HighMiB was eligible, in 8/31 patients LowMiR was eligible. In six out of these eight patients both HighMiB and LowMiR were eligible for direct comparison. In MR/CT perfusion imaging of the 16 patients eligible for HighMiB, 29/48 cortical regions of interest (ROIs) (60%) displayed hypoperfusion or ischemia, areas inadequately accessible by LowMiR. These ROIs made up 49% of the 59 ROIs displaying hypoperfusion or ischemia, altogether. Matching of parameters in normal and impaired ROIs between LowMiR and MRI/CT perfusion imaging was significantly poorer than in HighMiB.

**Conclusion:**

LowMiR using refill-kinetics potentially has the advantage of real time imaging and better resolution. The diagnostic impact, however, proves inferior to HighMiB both with respect to imaging quality and semi-quantitative evaluation.

## Introduction

In ultrasonic perfusion imaging (UPI), different methods are used to display parenchymal microvascular blood supply [1]. Two basic approaches are commonly applied in clinical settings. The bolus-kinetic approach analyses time-intensity curves that are derived in specific regions of interest (ROI) after a bolus injection of a standardized dose of contrast enhancer. The refill-kinetic approach additionally analyses noise alterations in specific ROIs. However, validation starts after the accomplishment of a steady state of contrast enhancer concentration in the blood pool with the destruction of contrast enhancer by the application of high mechanical index (MI) energy flashes followed by low MI sequences depicting the characteristic of replenishment (“refilling”) of contrast enhancer. In stroke patients, various reports have consistently proven the ability of characterizing ischemic areas. A reliable discrimination of critically hypoperfuzed and irreversibly ischemic areas (penumbra and core) has only been proposed for the high MI bolus-kinetics (HighMiB) approach [2–4].

Using two well-described specific UPI protocols, this study was designed to validate the potential of detecting the degree of impaired cerebral parenchymal perfusion. HighMiB with a bilateral approach and unilateral low MI refill-kinetics (LowMiR) were tested against timely matched perfusion-weighted MRI or perfusion CT in a group of patients with acute infarction caused by middle cerebral artery occlusion as seen in the preceding CTA or MRA eligible for interventional therapy.

## Materials and methods

### Patients

Patients with acute ischemic stroke admitted to the Stroke Center of the Inselspital, University Hospital Bern, Switzerland, between October 2012 and March 2014 who were scheduled for mechanical thrombectomy were enrolled in the study if an experienced sonographer (JE, MO, RR) was on duty. Enrollment was performed immediately after perfusion CT (CTP) or MR perfusion (MRP) imaging. UPI was performed after ultrasonic depiction of both MCAs in the angio-suite during the preparation for the interventional procedure and was not allowed to delay any therapeutic intervention. The sonographer was blinded to CT or MR perfusion results at the time of the examination. Patients with insufficient temporal bone window defined by the failure to depict standard landmarks were excluded [2]. Age below 18 and severe heart, lung and kidney diseases were defined as contraindication according to the regulatory rules of the echo-contrast agent SonoVue (Bracco Diagnostics, Milan, Italy). Severity of stroke was documented by use of the National Institutes of Health Stroke Scale on admission. The protocol for collecting data was in accordance with the 1996 revision (Somerset West, RSA) of the Declaration of Helsinki (1964) and was approved by the local ethics committee (Kantonale Ethikkommission Bern, No. KEK 179/12). Informed consent was obtained in all patients in a two-step procedure: (i) in the acute phase of the study, the patients´ relatives approved the participation of the patient in the study; (ii) in the follow-up phase, up to three months after stroke, the patients themselves approved, or, if aphasia was still present, the approval was obtained from the legal representative of the patient retrospectively. Written consent was mandatory under all circumstances.

### Ultrasound perfusion imaging

All examinations were performed using a Philips iU22 ultrasound machine (Philips Healthcare, Andover, MA, USA) equipped with a 1–5 MHz dynamic sector array (S5-1). The field-of-view (FOV) was set to an imaging depth of 150 mm in a sector angle of 90°. Peak systolic velocities (PSV) of both MCA were depicted. Imaging plane was then tilted to the diencephalic plane as described before [2], where the frontal horns of the side ventricles and the third ventricle serve as landmarks and where the MCA territory can be visualized well without major vessels producing artefacts.

HighMiB was defined as described before [2,4]. To enable evaluation of cortical ROIs, the probe was held contralaterally to the suspected symptomatic hemisphere. Data acquisition was obtained in of 45second periods immediately after iv injection of a 2.0 ml bolus of the contrast enhancer SonoVue (Bracco International, Milan, Italy) using an MI setting of 1.34 and a frame rate of 5 Hz. Data were then analyzed off-line using VueBox software version 4.3 (Bracco Imaging, Geneva, Switzerland) by fitting a bolus-model function and extracting specific parameters of perfusion [5]. Both region-wise analysis and that of calculated parameter images for the time-to-peak intensity (TPI) were performed [6]. ROI-wise analyses were performed in pre-specified ROIs with the analyses of unaffected hemisphere to achieve an intra-individual reference value for the depth independent time associated parameter TPI [6]. Evaluation of the affected hemisphere was performed by extracting these parameters in 10 pre-specified ROIs, including three cortical areas as well as three areas of the white matter, the basal ganglia (head of caudate nucleus and lentiform nucleus), and the thalamus. A TPI of >3 s was chosen as cut-off value for delayed perfusion (compare [4]), no perfusion was defined by missing rise of the time-intensity curve.

LowMiR was performed as described elsewhere [7] with depth of examination limited to 10cm and with the exception of extracting more specific parameters out of similarly placed ROIs in accordance to the high MI approach. This was done in order to gain as much as possible “spatial resolution”. Two data sets were taken without time delay from either hemisphere to enable the comparison of affected and unaffected areas. After bolus application of 2.5 ml SonoVue, data acquisition was started and the visualization of the contrast arrival using an MI of 0.17 with a frame rate of 16 Hz was gained. Immediately after that, nine flashes of high MI (0.87) were applied to destroy the microbubbles in the scanning plane, followed by 20 s of further data acquisition with the low MI settings for the detection of replenishment. Off line analysis was performed as described above by comparing the A and β values of the fitted replenishment-model and the product of Axβ in 7 pre-specified ROIs (caudate nucleus, anterior and posterior thalamus, lentiform nucleus, anterior / middle / posterior MCA white matter) using the VueBox software, version 4.3 (Bracco Imaging, Geneva, Switzerland). Insufficient temporal bone window was assumed once the low MI Bolus examination of the unaffected hemisphere did not display any signal.

### Magnetic resonance and computed tomography perfusion imaging (MRP/CTP) studies

CT/MRI perfusion was obtained in accordance to the standard-of-care protocol of the time and data were analyzed by three different investigators (RW, MO, RR) using a Food and Drug Administration (FDA) approved software (OleaSphere V2.3, OLEA Medical SA, La CiotatFrance). Analysis was performed using predefined circular ROIs of 50 mm^2^ (head of caudate nucleus, lentiform nucleus, anterior and posterior thalamus) and 200 mm^2^ (anterior, middle, and posterior MCA white matter as well as anterior, middle, and posterior MCA gray matter). For the quantification algorithm, the depicted ROIs were placed on a standard diencephalic plane as described previously [4], i.e. all ROIs are analyzed on the identical axial plane for both hemispheres. We defined the diencephalic plane by identification of lateral ventricles with their anterior and posterior horns, the plane crossing the third ventricle in its upper region surrounded by the thalamic and basal ganglia structures. The placement of the ROIs was performed after consensus of at least two of the three investigators (RW, MO, RR).

Time to peak (TTP) measurements of the ROIs on MRIs and CTs were extracted identically to the ROIs of the UPI investigation. The individual median of nonaffected contralateral ROIs served as reference and the cutoff threshold used was a delay of 4 s [4]. Categorization was done with a) “normal perfusion” = TTP within 4 s; b) “hypoperfusion” = TTP delay of >4 s, c) “no perfusion” = no rise of the time–intensity curve detectable. For the categorization of relative cerebral blood flow (rCBF) and relative cerebral blood volume (rCBV) thresholds were used as described before [8], i.e. 0.67 and 0.74 respectively.

### Analyses and statistical evaluation

Descriptive statistics (mean, median, s.d. and percentiles) were applied for all UPI parameters (TPI for HighMiB and A, β, and Axβ for LowMiR) and perfusion MRI/CT parameter (TTP, MTT, rCBF and rCBV). Normal distribution was shown for the UPI parameters TPI and for the MRP/CTP parameter MTT and rCBF, whereas all the other parameter had no normal distribution. Statistical analyses were done using the SPSS Statistics Software (Version 22, Chicago, IL, USA).

Further evaluation was three-split:

1. First, correlation of LowMiR values of rise rate (β) and plateau (Axβ) with MRP/CTP values of rCBV and rCBF was tested by using a Bland-Altman plot.
2. Second, correlation of HighMiB values and MRP/CTP has already been described and partially been published elsewhere [4]; the HighMiB data of the 16 patients have been compared to MRP/CTP. Analysis of receiver operating characteristics proved a high sensitivity of HighMiB in the diagnosis of hypo perfused (AUC = 0.917; p <0.001) and non-perfused (AUC = 0.830; p <0.001) tissue in comparison with CTP and MRP. Therefore, analyses have not been repeated here. Here, we retested these values by plotting the individual TTP delay of each ROI of the affected hemispheres as to the mean of ROIs of the same patients with the according MRP/CTP values.
3. Third, LowMiR (β and Axβ) and HighMiB (TTP delay) parameters of those six patients in which both methods could be successfully performed were dichotomized according to the previously described threshold values of pathological perfusion for the according parameters. These were for β < 0.76, for Axβ < 1.91 in LowMiR [7], and for TTP > 3s in HighMiB [4]. ROIs were then categorized in a fourfold table depending on the accordingly dichotomized parameter of rCBF/rCBV or TTP in MRP/CTP with a threshold of 0.67/0.74 or 4 s for direct qualitative comparison of both UPI methods with CT-/MRP by displaying positive and negative predictive values of the detection of any perfusion impairment. Only those ROIs were chosen, which were eligible for analysis both with HighMiB and LowMiR, i.e. cortical ROIs were not included.

## Results

### Study population

**Fig 1:**
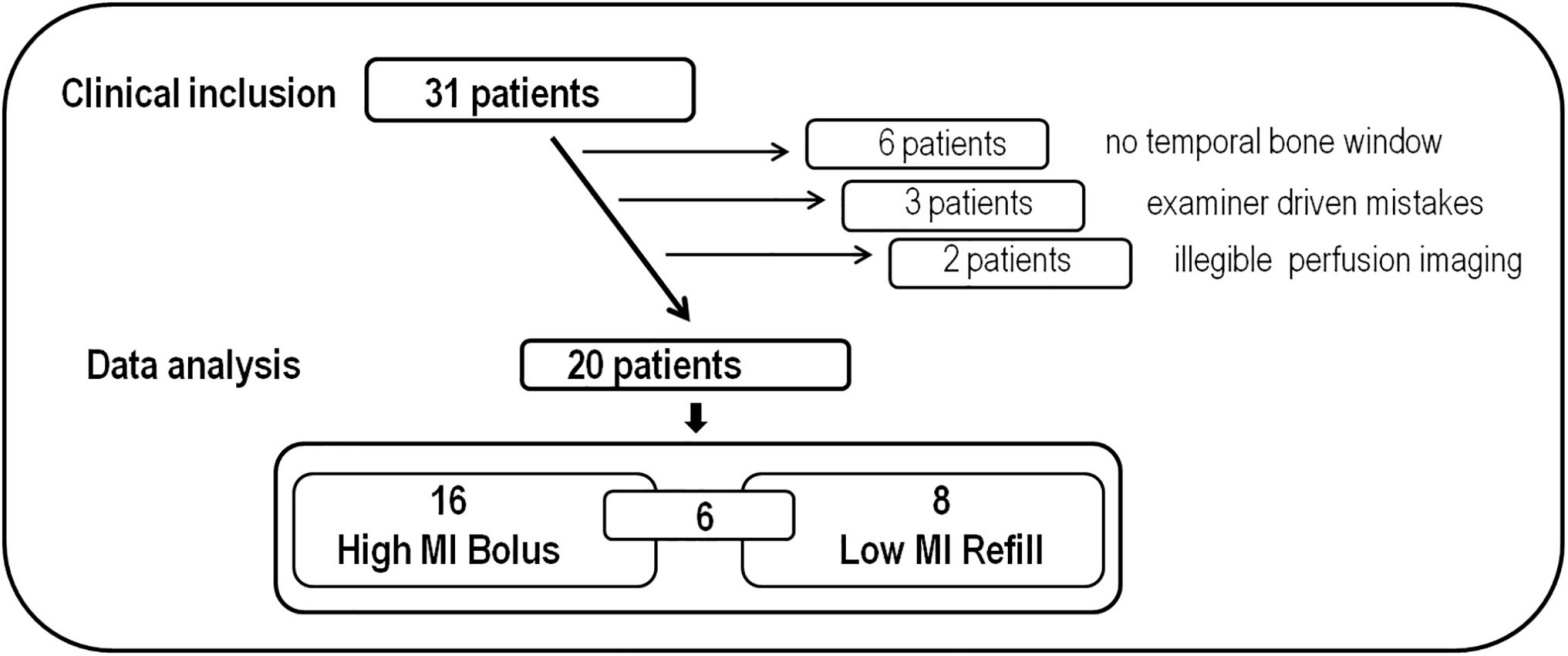
Flow of patients´ recruitment.

Thirty-one consecutive patients were included (see Table 1). Six patients (19.4%) did not display a sufficient temporal bone window. In three patients, examiner driven shortcomings led to illegible ultrasound examinations. In two patients, the perfusion weighted standard examination (CT or MRI) was not eligible. Therefore, data of only 20 patients (64.5%) with at least one sufficient temporal bone window were eligible for further analyses. In 16 patients (51.6%), HighMiB was technically successful; in 10 patients (32.3%), LowMiR was eligible, in two of which only the non-affected side was eligible. In six (19.4%) out of the remaining eight patients, both HighMiB and LowMiR were successful (see Fig 1).

**Table 1:** Patients´ demographic data.

Consecutive patient No.	Occluded branch	Age	Sex	NIHSS admission	SOT [min]	HighMiB	LowMiR	CTP/MRP
**1**	rM1	76	F	15	123	Y	N	MRP
**2**	rM1	93	M	17	63	N	N	CTP
**3**	lICA	72	F	6	245	Y	N	MRP
**4**	lICA	81	M	17	55	Y	N	MRP
**5**	lM1	90	F	20	45	N	N	CTP
**6**	rM1	75	F	8	90	Y	N	MRP
**7**	rM1	80	F	4	278	Y	N	CTP
**8**	rICA, rM1	70	M	13	85	Y	Y	MRP
**9**	lM1	86	M	24	50	Y	Y	CTP
**10**	rICA, rM1	79	F	17	40	N	N	CTP
**11**	lM1	81	M	28	90	N	N	CTP
**12**	rM1	78	F	11	255	Y	N	MRP
**13**	lICA-T	65	F	21	289	N	N	CTP
**14**	rM2	70	M	8	152	N	N	MRP
**15**	rM1	85	F	7	100	Y	N	MRP
**16**	rM1	80	F	15	53	N	N	MRP
**17**	lM2	65	M	3	77	Y	N	MRP
**18**	rM1	63	F	7	WUS	N	N	MRP
**19**	lICA-T	49	F	19	WUS	N	N	CTP
**20**	rICA	80	F	14	195	Y	N	MRP
**21**	lM2	68	F	11	WUS	N	N	MRP
**22**	rM1	56	M	7	74	N	Y	CTP
**23**	rM1	75	M	16	166	N	Y	CTP
**24**	lM2	73	F	7	52	Y	N	MRP
**25**	rM2	74	F	5	121	Y	Y	MRP
**26**	rICA, rM3	74	M	25	132	N	N	MRP
**27**	rICA, rM1	80	F	19	256	N	N	CTP
**28**	lM4	76	M	7	192	Y	Y	MRP
**29**	lM3	74	M	3	63	Y	Y	MRP
**30**	rM1	36	M	3	218	N	N	CTP
**31**	lM1	79	M	20	46	Y	Y	MRP
**Mean/sum**		**74**	**17 F**	**13**	**129**	**16**	**8**	
**SD**		**11**		**7**	**81**			

Demographic data of patients included in the study. Side of occlusion: r = right, l = left. Occluded vessel: M = middle cerebral artery segments 1–4; ICA = internal carotid artery; ICA-T = t-occlusion. SOT = symptom to onset time (if known). WUS = wake up stroke (if not known). Kinetics: HighMiB = high mechanical index bolus, LowMiR = low mechanical index refill. Ultrasound perfusion examination: Y = successful, N = not successful. Mode of standard perfusion imaging: CTP = CT perfusion, MRP = MR perfusion.

Mean age of the 31 patients was 74 yrs (±11) with 17 female patients and with 3 "wakeup strokes" with unknown symptom onset and a mean "symptom to onset time" (SOT) of 129 min (±81) in the other 28 patients with a mean NIHSS score of 13 (±7). About half of the patients (except pat. no. 3,5,6,8,10,11–13,16,18–22,30) had running systemic thrombolysis with a delay between symptom onset and start of thrombolysis of 157 min (±49). The delay between either MRP or CTP to actual start of UPI was 58 min (±36). Side effects of the ultrasound contrast enhancer were detected in no patient.

In the 16 patients with successful HighMiB (age 77±11 yrs, 9 female, SOT 127±80 min, NIHSS score 10±6), 14 MRP and 2 CTP exams were undertaken before UPI. Eight patients had right-sided stroke with mostly proximal occlusion in the M1 segment in 44%, ICA occlusion in 25%, and M2/3/4 occlusion in 19/6/6%, respectively.

In the eight patients with successful LowMiR (age 74±9 yrs, 1 female, SOT 100±55 min, NIHSS score 12±8), five MRP and three CTP exams were undertaken before UPI. Three patients had right-sided stroke with mostly proximal occlusion in the M1 segment in 4/8, and ICA as well as M2/3/4 occlusion in 1/8.

In the six patients with both successful LowMiR and HighMiB (age 77±6 yrs, 1 female, SOT 93±56 min, NIHSS score 12±9), five MRP and one CTP exams were undertaken before UPI. Two patients had right-sided stroke with most proximal occlusion in the M1 segment in three patients, and ICA as well as M2/3/4 occlusion in one patient.

### Perfusion deficits as defined by reference standard

For data values of perfusion imaging modalities see Tables 2–4. Perfusion deficits in those 16 patients eligible for HighMiB were detected in MRP/CTP in 59/160 (39%) ROIs with 29 ROIs being located in cortical areas (49% of all pathological ROIs). None of the ROIs with impaired perfusion was categorized as “no perfusion”, whereas the rest was categorized as “delayed perfusion”.

**Table 2:** Perfusion imaging values of patients 8, 9, and 22, eligible for LowMiR imaging.

Patient	8									9									22						
*mode*	*HighMiB*	*MRP*			*LowMiR*			*MRP*		*HighMiB*	*CRP*			*LowMiR*			*CTP*				*LowMiR*			*CTP*	
value	TTP	TTP	A	MTT	β	Axβ	MTT	CBF	CBV	TTP	TTP	A	MTT	β	Axβ	MTT	CBF	CBV	A	MTT	β	Axβ	MTT	CBF	CBV
**reference**																									
ipT	23,72	31,20	128,00	0,30	3,33	426,67	17,74	26,78	7,35	16,97	21,57	0,60	13,24	0,08	0,05	7,22	9,41	1,03	0,67	0,94	1,06	0,63	10,06	40,65	4,85
iaT	23,03	28,18	40,00	0,34	2,94	117,65	19,04	28,51	8,35	15,65	20,68	0,49	1,93	0,52	0,25	8,53	15,06	2,19	na	na			10,44	22,35	5,57
iLN	22,89	26,42	160,00	0,12	8,33	1333,33	4,96	22,54	2,05	na	22,16	1,31	14,50	0,07	0,09	7,79	9,50	1,28	0,61	0,59	1,69	0,36	7,60	19,85	2,44
iCN	22,23	26,19	16,00	0,23	4,35	69,57	4,58	24,48	1,46	na	21,49	0,89	2,36	0,42	0,38	7,44	16,45	1,59	na	na			10,55	40,97	5,91
mean	22,97	28,00								16,31	21,48														
SD	0,53	2,00								0,66	0,53														
median	22,96	27,30								16,31	21,53														
first quartile	22,73	26,36								15,98	21,29														
3rd quartile	23,20	28,94								16,64	21,72														
quartile deviation	0,24	1,29								0,33	0,22														
**affected**																									
cCN	25,92	27,59	17,22	1,09	0,92	15,80	4,96	28,02	2,18	na	15,62	0,42	1,83	0,55	0,23	3,43	2,38	0,30	4,40	9,10	0,11	40,04	9,34	43,50	3,00
caT	21,67	28,24	52,39	1,38	0,72	37,96	8,21	38,83	6,24	16,40	21,55	4,02	5,56	0,18	0,72	0,73	14,44	1,81	3,41	9,70	0,10	33,08	9,60	28,43	4,02
cpT	24,74	28,18	303,64	3,29	0,30	92,29	10,33	24,46	4,41	16,80	21,73	0,94	0,81	1,23	1,16	8,11	19,11	2,43	8,45	18,02	0,06	152,27	8,75	22,38	2,87
cLN	24,38	29,44	41,31	1,12	0,89	36,88	7,74	18,23	2,05	12,15	26,13	6,68	3,71	0,27	1,80	6,53	0,82	5,12	16,56	11,22	0,09	185,80	8,84	21,08	2,87
cWMa	26,92	64,83	0,18	12,8	0,08	0,01	20,04	10,72	2,46	np	32,48	0,34	3,12	0,32	0,11	6,80	4,35	0,51	50,31	11,74	0,09	590,64	7,01	19,84	4,25
cWMm	np	60,70	0,01	0,26	3,85	0,04	11,66	3,79	0,90	np	28,66	3,84	15,60	0,06	0,25	7,36	3,80	0,44	28,11	10,25	0,10	288,13	8,70	33,42	4,24
cWMp	np	58,38	1,63	28,2	0,04	0,06	15,18	6,46	1,76	np	32,24	2,80	90,68	0,01	0,03	7,86	8,42	1,21	18,33	43,30	0,02	793,69	7,80	30,19	3,74
cCora	np	76,25	x	x	x	x	15,89	3,85	1,24	np	29,11	x	x	x	x	8,51	8,05	1,18	x	x	x	x	8,24	22,55	3,00
cCorm	np	63,31	x	x	x	x	11,19	6,77	1,13	np	25,26	x	x	x	x	7,36	3,80	0,44	x	x	x	x	6,35	16,52	2,13
cCorp	np	57,66	x	x	x	x	17,10	8,63	2,50	np	31,89	x	x	x	x	7,46	12,59	1,60	x	x	x	x	6,59	19,84	2,47

HighMiB = high mechanical index bolus approach; MRP = magnetic resonance perfusion imaging; LowMiR = low mechanical index refill approach; TTP = time to peak intensity; A = A value; β = β value; MTT = mean transit time; CBF = cerebral blood flow; CBV = cerebral blood volume; ipT = ipsilateral posterior thalamus; iaT = ipsilateral anterior thalamus; iLN = ipsilateral lentiform nucleus; iCN = ipsilateral caudate nucleus; cCN = contralateral caudate nucleus; caT = contralateral anterior thalamus; cpT = contralateral posterior thalamus; cLN = contralateral lentiform nucleus; cWMa = contralateral anterior white matter; cWMm = contralateral middle white matter; cWMp = contralateral posterior white matter; cCora = contralateral anterior cortical area; cCorm = contralateral middle cortical area; cCorp = contralateral posterior cortical area.

**Table 3:** Perfusion imaging values of patients 23, 25, and 28, eligible for LowMiR imaging.

Patient	23							25									28								
*mode*			*LowMiR*			*CTP*		*HighMiB*	*CTP*			*LowMiR*			*CTP*		*HighMiB*	*MRP*			*LowMiR*			*MRP*	
value	A	MTT	β	Axβ	MTT	CBF	CBV	TTP	TTP	A	MTT	β	Axβ	MTT	CBF	CBV	TTP	TTP	A	MTT	β	Axβ	MTT	CBF	CBV
**reference**																									
ipT	14,89	1,77	0,56	8,41	6,14	18,15	1,53	31,18	28,07	79,00	0,51	1,96	154,90	2,19	31,18	1,61	19,45	23,12	2,99	2,95	0,34	1,01	2,13	19,27	1,51
iaT	15,85	2,31	0,43	6,86	6,23	14,30	2,60	30,48	28,19	29,63	1,43	0,70	20,72	10,87	104,76	19,14	19,65	23,52	2,08	5,87	0,17	0,35	5,92	38,89	3,10
iLN	46,59	1,28	0,78	36,40	5,42	15,40	1,53	29,97	27,09	54,00	0,99	1,01	54,55	1,16	28,82	0,65	20,03	23,44	26,43	0,87	1,15	30,38	11,38	16,49	1,39
iCN	33,51	2,47	0,40	13,57	6,38	17,94	2,00	29,29	26,82	9,04	19,13	0,05	0,47	3,68	35,16	1,99	19,69	22,62	2,02	4,14	0,24	0,49	6,08	20,97	1,68
mean								30,23	27,54								19,71	23,18							
SD								0,69	0,60								0,21	0,35							
median								30,23	27,58								19,67	23,28							
first quartile								29,80	27,02								19,60	23,00							
3rd quartile								30,66	28,10								19,78	23,46							
quartile deviation								0,43	0,54								0,09	0,23							
**affected**																									
cCN	174,00	4,00	0,25	43,50	6,46	21,70	2,00	31,86	27,26	1,34	1,04	0,96	1,29	2,10	48,67	1,67	20,11	22,50	13,45	1,79	0,56	7,51	8,51	17,38	1,69
caT	33,50	2,10	0,48	15,95	5,76	13,40	3,64	32,24	27,83	5,58	0,21	4,76	26,57	10,73	65,91	11,37	20,01	23,97	3,72	0,67	1,49	5,55	4,53	39,08	2,81
cpT	35,70	2,40	0,42	14,88	7,47	29,27	3,64	31,72	28,38	11,09	2,30	0,43	4,82	6,19	32,26	3,08	22,29	23,54	1,10	1,39	0,72	0,79	2,29	24,31	2,10
cLN	62,00	2,30	0,43	26,96	7,32	21,56	2,49	31,52	27,59	11,26	1,53	0,65	7,36	4,17	51,11	3,43	20,05	22,47	2,35	1,07	0,93	2,20	8,17	16,62	1,54
cWMa	51,62	0,28	3,57	184,36	6,44	18,22	1,91	29,43	27,56	0,24	7,71	0,13	0,03	1,54	45,31	1,48	20,41	22,85	9,96	0,11	9,09	90,55	5,36	35,30	2,62
cWMm	32,44	1,38	0,72	23,51	4,30	19,19	1,63	31,70	29,14	11,74	1,75	0,57	6,71	4,02	33,74	1,69	19,49	23,04	5,42	0,17	5,88	31,88	5,90	23,27	1,89
cWMp	29,35	10,57	0,09	2,78	6,23	9,92	1,03	32,58	38,87	0,22	1,08	0,93	0,20	6,00	49,59	4,26	22,23	22,92	5,40	0,38	2,63	14,21	4,70	29,92	2,35
cCora	x	x	x	x	6,78	15,33	1,45	30,80	27,50	x	x	x	x	1,97	34,74	1,32	20,51	22,67	x	x	x	x	4,40	36,90	2,63
cCorm	x	x	x	x	7,60	15,69	1,91	36,80	28,71	x	x	x	x	4,88	27,11	2,34	19,61	23,37	x	x	x	x	2,68	19,78	1,71
cCorp	x	x	x	x	5,07	18,23	1,50	33,60	37,09	x	x	x	x	10,90	69,02	10,06	18,81	23,41	x	x	x	x	5,21	31,28	2,33

**Table 4:** Perfusion imaging values of patients 31 and 12, eligible for LowMiR imaging.

Patient	31									12								
*mode*	*HighMiB*	*MRP*			*LowMiR*		*MRI*			*High MiB*	*MCTP*			*LowMiR*			*CTP*	
value	TTP	TTP	A	MTT	β	Axβ	MTT	CBF	CBV	TTP	TTP	A	MTT	β	Axβ	MTT	CBF	CBV
**reference**																		
ipT	27,81	30,97	0,78	17,65	0,06	0,04	1,24	20,17	0,47	21,73	26,61	no TIC	no TIC	no TIC	no TIC	2,32	128,80	5,74
iaT	26,73	30,43	0,74	25,21	0,04	0,03	3,35	26,33	1,49	22,85	27,24	no TIC	no TIC	no TIC	no TIC	3,05	137,99	7,26
iLN	26,57	31,22	4,37	6,30	0,16	0,69	5,82	7,37	0,88	20,89	22,91	no TIC	no TIC	no TIC	no TIC	1,33	70,10	2,40
iCN	25,97	31,16	1,51	8,92	0,11	0,17	11,62	13,22	2,03	20,21	26,61	no TIC	no TIC	no TIC	no TIC	2,96	89,41	4,86
mean	26,77	30,95								21,42	25,84							
SD	0,66	0,31								0,99	1,71							
median	26,65	31,07								21,31	26,61							
first quartile	26,42	30,84								20,72	25,69							
3rd quartile	27,00	31,18								22,01	26,77							
quartile deviation	0,29	0,17								0,65	0,54							
**affected**																		
cCN	27,69	32,10	0,55	92,75	0,01	0,01	11,51	14,86	3,32	na	28,20	3,10	0,30	3,33	0,93	3,36	42,84	3,26
caT	27,53	29,91	1,78	9,46	0,11	0,19	2,98	12,47	0,88	21,45	30,77	13,51	1,59	0,63	21,48	3,11	93,09	4,81
cpT	28,87	31,01	1,57	8,76	0,11	0,18	0,00	13,17	0,00	24,11	30,39	33,61	3,14	0,32	105,54	2,49	81,61	4,86
cLN	28,23	24,87	3,80	1,05	0,95	3,62	0,03	7,74	0,00	np	42,63	40,08	5,79	0,17	232,06	1,90	13,81	0,44
cWMa	27,63	34,78	3,26	5,72	0,17	0,57	5,77	16,23	1,75	np	50,05	0,82	39,62	0,03	32,49	11,06	15,19	2,67
cWMm	29,79	44,69	4,16	23,98	0,04	0,17	17,69	11,14	3,13	21,50	33,11	16,25	1,82	0,55	29,58	2,48	54,18	2,98
cWMp	30,70	47,08	3,22	3,26	0,31	0,99	10,04	14,57	2,89	18,19	32,53	5,70	4,50	0,22	25,65	5,30	99,61	7,01
cCora	33,60	35,67	x	x	x	x	7,28	21,53	4,02	np	30,26	x	x	x	x	4,66	75,91	5,35
cCorm	31,84	50,02	x	x	x	x	17,25	15,63	4,18	np	34,50	x	x	x	x	5,48	40,55	4,11
cCorp	29,70	49,54	x	x	x	x	19,50	12,16	4,02	25,41	34,17	x	x	x	x	4,08	52,93	4,16

### Perfusion deficits as defined by UPI

**Fig 2:**
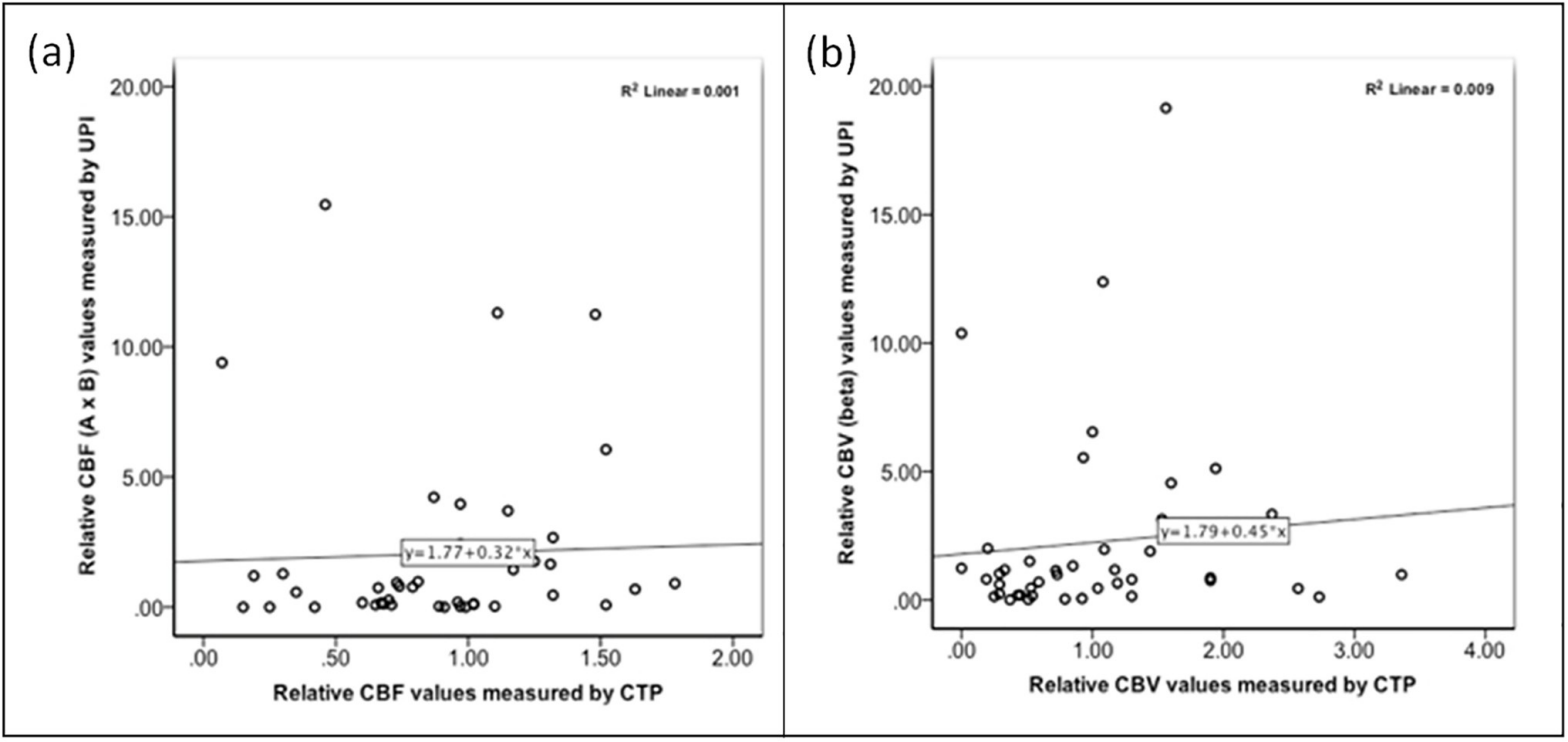
Scatter plot for correlation between LowMiR and CT/MRI rCBF. Scatter plot showing a poor correlation between Axβ (a) and β (b) values measured by LowMiR UPI versus perfusion CT/MRI rCBF (a) and rCBV (b). R^2^ coefficient of determination is 0.001 (a) and 0.009 (b), respectively.

**Fig 3:**
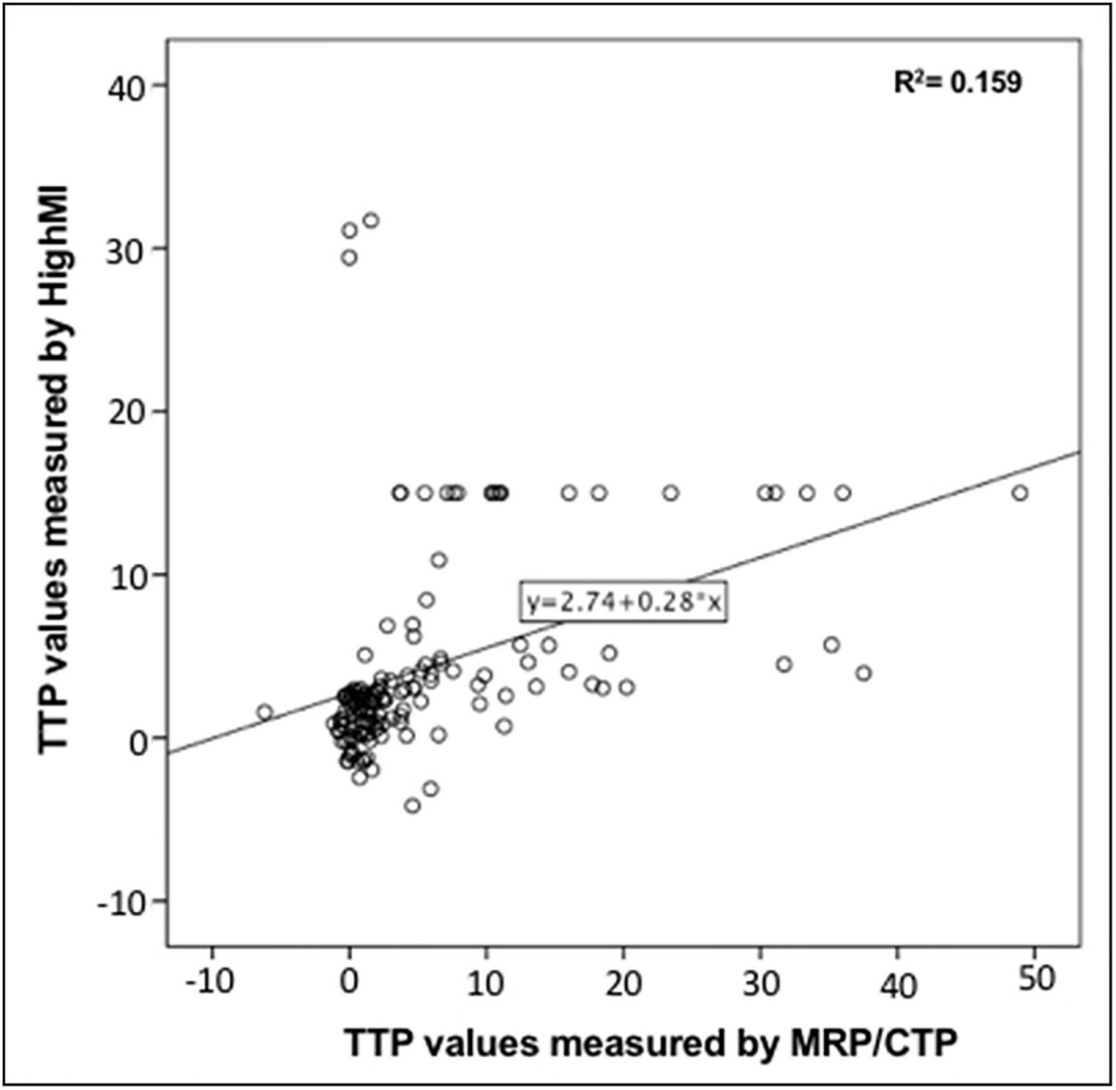
Scatter plot for correlation between HighMiB and CT/MRI TTP values. Scatter plot showing the linear correlation between absolute TTP delay values measured by HighMiB UPI versus perfusion CT/MRI. R^2^ coefficient of determination is 0.159.

**Fig 4:**
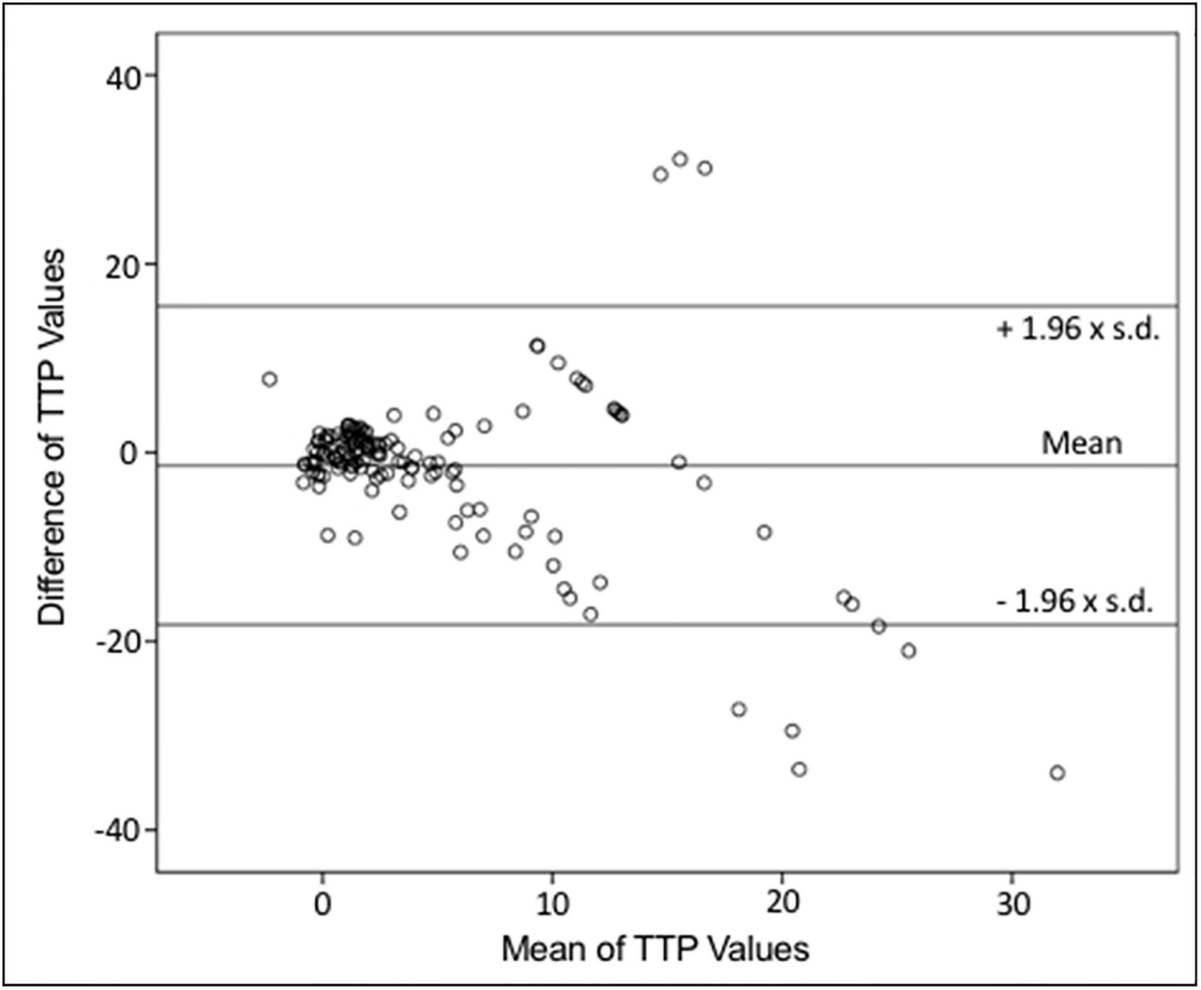
Bland Altman plot for mean of TTP values as measured by HighMiB Investigation in comparison to MRP/CTP.

For data values of UPI imaging modalities see Figs 2 and 3, as well as Tables 5–7. LowMiR analyses displayed a wide range of A, ß, and Axß. Statistical analyses using linear regression analyses revealed poor correlation of ß to rCBV (of MRI/CT perfusion imaging) and of Axß to rCBF in areas categorized as “delayed” or “non-perfused” as seen in Fig 2. ROC analyses of HighMiB TPI values have formerly shown high sensitivity of ultrasound perfusion imaging in the diagnosis of hypo-perfused area under the curve, (AUC = 0.917; p<0.001) and nonperfused (AUC = 0.830; p<0.001) tissue in comparison with MRP/CTP [4]. Here, we analyzed the correlation of the TPI / TTP delay of HighMiB and CTP/MRP, respectively, also using scatter plot revealing linear correlation as seen in Fig 3. Bland Altman plot showed the correlation between analyzes done with HighMiB versus MRP / CTP (Fig 4). Differences for TTP measured by HighMI in comparison to MRP/ CTP examinations were on average -1.36±0.71, resulting in a coefficient of repeatability of 16.87. By trying to apply the Bland Altman test for the ß versus rCBV or Axß versus rCBF analyses, the difference between the values proved to be significant, so that these values cannot show a level of agreement and a Bland-Altman test was not feasible. Indirect comparison of the diagnostic accuracy in the six patients with both UPI modalities (i.e., LowMiR vs. HighMiB) displayed a PPV and NPV values as displayed in Tables 3 to 5.

**Table 5:** Four-fold table for the analysis of PPV and NPV of HighMiB TTP in comparison to CTP/MRP.

TTP	CTP/MRP positive	CTP/MRP negative
**HighMiB positive**	8	0
**HighMiB negative**	4	48

PPV and NPV values of ROIs categorized as positive by HighMiB TTP >3 s in comparison to CTP/MRP defined positive with a pathological perfusion (TTP >4 s) in 66 ROIs. Note that cortical ROIs were not included and that in six ROIs HighMiB analyses were not applicable. PPV = 1. NPV = .92

**Table 6:** Four-fold table for the analysis of PPV and NPV of LowMiR β in comparison to CTP/MRP.

Β	CTP/MRP positive	CTP/MRP negative
**LowMiR positive**	0	39
**LowMiR negative**	0	23

PPV and NPV values of ROIs categorized as positive by LowMiR β <0.76 in comparison to CTP/MRP defined positive with a pathological perfusion (TTP >4 s) in 66 ROIs. Note that cortical ROIs were not included and that in 4 ROIs LowMiR analyses were not applicable. PPV = 0. NPV = 1

**Table 7:** Four-fold table for the analysis of PPV and NPV of LowMiR Axβ in comparison to CTP/MRP.

Axβ	CTP/MRP positive	CTP/MRP negative
**LowMiR positive**	6	28
**LowMiR negative**	2	26

PPV and NPV values of ROIs categorized as positive by LowMiR Axβ <1.91 in comparison to CTP/MRP defined positive with a pathological perfusion (TTP >4 s) in 66 ROIs. Note that cortical ROIs were not included and that in 4 ROIs LowMiR analyses were not applicable. PPV = .18. NPV = .63

## Discussion

**Fig 5:**
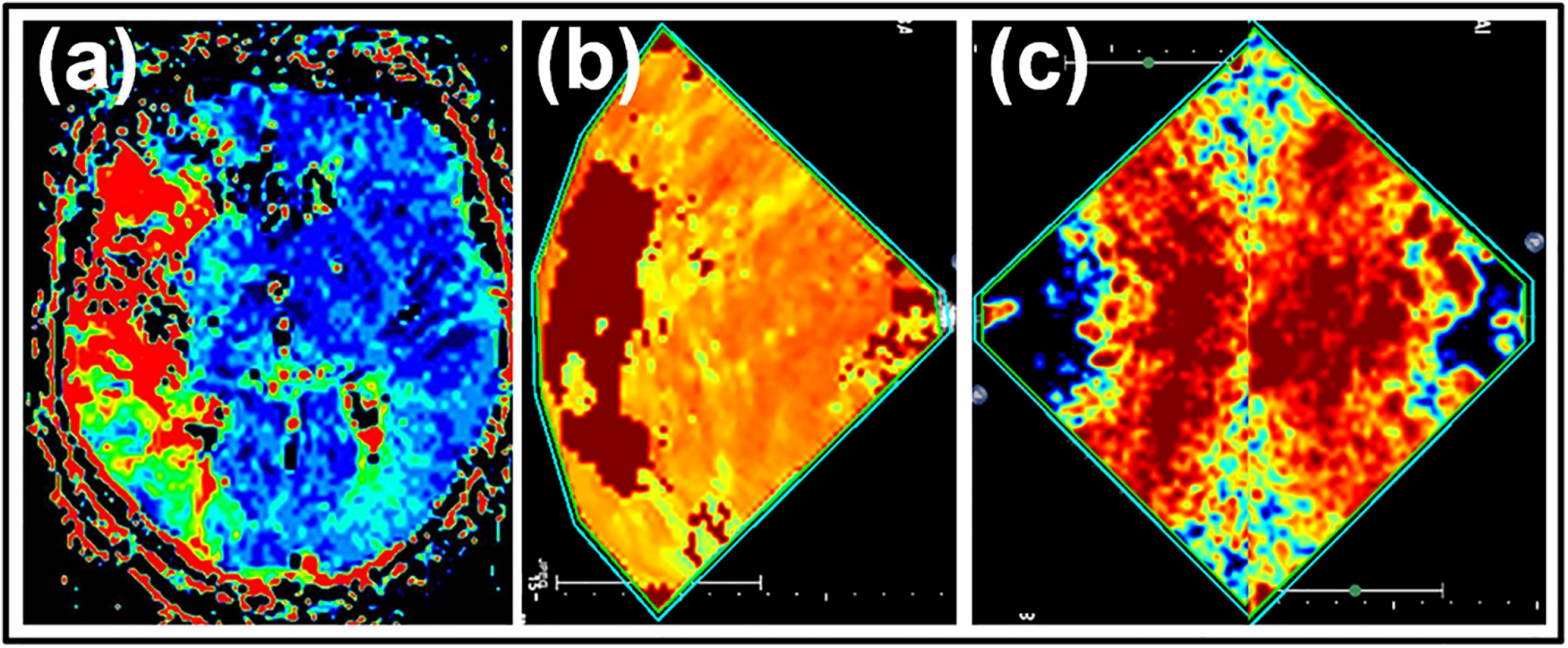
Parameter maps of perfusion imaging in patient 8 (occlusion of M1 branch of MCA). (a): MRP-TPI parameter map displaying perfusion deficit in >2/3 of the right MCA territory; (b): HighMiB-TPI parameter map tilted in accordance to MRP orientation taken from the side contralateral to the expected perfusion lesion displaying a perfusion deficit in the according area; (c): LowMiR-rCBV parameter maps taken from both sides and tilted/overlaid according to MRP orientation displaying the restricted depth of insonation (evaluation restricted to the hemisphere close to the probe) and the restricted area of possible quantification due to the near filed artifacts in the non-affected hemisphere.

UPI is a technique of micro-vascular, parenchymal imaging that has been introduced in the late 1990s. Different ultrasound techniques and modalities of data acquisition have since been validated [1,9,10]. Since CT perfusion imaging has since been widely established as a standard imaging technique, the potential advantages of mobility and easy repeatability of ultrasound imaging have been lost sight of in acute stroke imaging. Using the so-called bilateral approach with the affected hemisphere in the far field, the non-affected hemisphere can be used as an intra-individual reference for specific parameters (such as the TTP) with the bolus-kinetics approach. Hereby, areas of the basal ganglia, white matter, and cortical regions can be evaluated [2,4]. Due to the settings used in low MI imaging, insonation parameters only enable evaluation up to a depth of about 10 cm. Therefore, low MI imaging has technically to be performed from the affected side (unilateral approach); hence, those structures closest to the probe (cortical areas), are lost for evaluation due to well-known technical near-field artifacts (compare Fig 5). In our series of consecutive stroke patients, the considerable amount of 49% of the affected ROIs (as defined by MRP/CTP) were located in cortical areas, which can be attributed to the expected rate of territorial ischemia due to middle cerebral artery occlusion in a series of acute stroke. HighMiB, performed from the side of the unaffected hemisphere, can evaluate these ROIs with good correlation to MRP or CTP [4]. However, HighMiB examinations are limited to one plane of insonation per time. In bolus tracking, a certain amount of time (of about 15–20 min) is necessary for the bolus to “decay” before the next examination can be taken. The refill-kinetic approach, on the other hand, enables numerous examinations in various planes of insonation [7]. Since the contrast agent in the blood pool practically stays saturated after infusion of the contrast agent for some minutes, multiple examinations in several planes are possible in a certain time frame. Therefore, this approach has also been termed as a “real-time” examination. Since the discrimination of the ischemic core of infarction from functionally impaired hypo-perfused tissue (penumbra) is of special interest in acute stroke (e.g., once considering revascularizing therapies), a comparison of both methods in “real life” under clinical settings is of substantial interest and therefore challenged here. If the examination quality were the same between methods, the “real-time” aspect would be, indeed, an advantage.

However, in our series we found some substantial drawbacks of both methods. Only in 18 out of 31 patients consecutively included, any UPI method displayed evaluable data. This may be attributable to a great extent to the emergency setting in the angio-suite just before interventional therapy. Therefore, the main result of our study is that UPI using current techniques cannot be regarded as a sufficient diagnostic tool in an emergency setting despite its´ inherent advantages of mobility as a bed-site technique. LowMiR displayed evaluable data in only half of the patients in which HighMiB was possible. Furthermore, quantification of perfusion parameters was not as satisfactory as might have been expected from the literature. Kern and colleagues [7] found semi-quantitative perfusion parameters in their collective (up to **48 hrs** after symptom onset, though) using the LowMiR approach. The β value (as a parameter of rise rate or rCBV) was found to be diminished in the area of stroke, the mean β ratio ischemic/normal was 0.48. The product of Axβ (as a function of rCBF) and A (as a parameter of plateau) were lower in ischemia. A good correlation was only found between the ischemic and normal hemispheres; however, the only form of quantification was possible using the rtTTP (i.e. bolus-kinetics using a low MI with above mentioned restricted depth of examination). Furthermore, ROIs were placed in the affected hemisphere without standardized size and localization. In our collective, we were able to reproduce the insufficient nature of cerebral perfusion quantification using the low MI refill-kinetics approach. At the same time, high MI bolus tracking proved to consistently well reproduce perfusion impairments as displayed by golden standard MRP or CTP.

LowMiR using refill-kinetics potentially has the advantage of real time (multi-plane) imaging and better resolution, whereas it has previously been discussed that HighMiB has the disadvantage of a so-called shadowing effect, implying that due to the burst of destructed microbubbles the areas further from the probe should not be evaluable. In this trial, however, we were able to show in accordance to former studies, that the HighMiB approach is capable of detecting perfusion deficits throughout both hemispheres. Furthermore, technical progress will allow multidimensional data acquisition in the near future, so that bolus tracking will also be possible in a multi-plane manner. Thus, if quantification is mandatory, the high MI bolus approach delivers more robust data.

There are some further limitations to this unblinded study, the most striking one is the small sample size as mentioned above. For direct comparison of the two approaches, data of only 6 patients could be used. This is partly due to the strikingly high number of technically unsuccessful examinations using the low MI approach. Only in 8/20 patients the LowMiR exam was technically successful, whereas in 16/20 patients HighMiB exams displayed evaluable data. Statistical analysis and conclusions are limited due to the small amount of only 6 patients evaluable for direct comparison of the two methods. However, within these six patients, 66 ROIs were used for analysis, because of which statistical analyses were performed as described. Another limitation is the heterogeneous character of our cohort. All patients with a reported MCA occlusion were included, resulting in a range of patients with a distal carotid T- and M1 occlusion up to patients with a M3/4 branch occlusion in final angiography. Furthermore, comparisons of both UPI techniques were drawn to either CT- or MR-perfusion, somewhat impeding the congruency of the results. However, the study was designed to proof the principle feasibility of ultrasound perfusion imaging in the complex situation of acute diagnosis and treatment in stroke patients, and, therefore, heterogeneous vessel and perfusion pathology was rather aimed for.

## Conclusion

Ultrasound imaging inherits the advantage of mobile application and could, therefore, theoretically be used in a preclinical setting as an additional multimodal diagnostic tool to guide early stroke treatment, detecting occlusion of large vessels [11], ruling out intracerebral hemorrhage [12], and displaying parenchymal perfusion status. It has not yet been proven which of the established perfusion approaches would serve best. In this small series, HighMiB proves the higher potential for differentiating ischemic and hypoperfuzed tissue both with respect to imaging quality and semi-quantitative evaluation. Therefore, the potential of multimodal ultrasound imaging using high MI bolus tracking should now be tested in a larger series in a blinded and multicenter approach. For the time being, with all limitations seen in the emergency setting this study, UPI might serve as a potential diagnostic tool for early follow up examinations in patients undergoing revascularizing therapies in ischemic stroke.

## Supporting information

S1 Dataset Dataset of Tables 2–4 containing all original data concerning the direct comparison of the two UPI groups. (XLSX)

S2 Dataset Containing data of HighMiB examinations of those patients where LowMiB was not successful for direct comparison (patients 1,3,4,6,7,15,17,20,24). (XLSX)
